# Surgical management of adenocarcinoma of the transverse colon: What should be the extent of resection?

**DOI:** 10.1002/ags3.12380

**Published:** 2020-07-26

**Authors:** Manas K. Roy, Amrit Pipara, Ashok Kumar

**Affiliations:** ^1^ GI‐HPB Surgery Unit Tata Medical Centre Kolkata India; ^2^ Department of Surgical Gastroenterology Sanjay Gandhi Postgraduate Institute of Medical Sciences Lucknow India

**Keywords:** adenocarcinoma, cancer, extent of resection, surgery, transverse colon

## Abstract

Transverse colon, owing its origin to midgut and hindgut and harbouring a flexure at both ends, continues to pose a surgical challenge. When compared to the rest of the colon, transverse colon adenocarcinoma is relatively uncommon. These cancers usually present late and lie in close proximity to the stomach, omentum, and pancreas. Adequate lymphadenectomy entails dissection around and ligation of the middle colic vessels. Hence, resectional surgery for transverse colon carcinoma is considered difficult. This is more so because of the variation of arterial and venous anatomy. From this perspective, the surgeon is tempted to perform a more radical operation like extended right or left hemicolectomy to secure an adequate lymphadenectomy. Such a cancer has also been dealt with a more limited transverse colectomy with colo‐colic anastomosis. For all these reasons, patients with transverse colon adenocarcinoma were excluded from randomised trials which compared laparoscopic resection with traditional open operation. Surgical literature is yet to establish a definite operation for transverse colon cancer and the exact procedure is often dictated by surgeon's preference. This is primarily because this is an uncommon cancer. The rapid adoption of laparoscopic operation favoured extended colectomy as transverse colectomy can be difficult by minimally invasive technique. However, in the recent past, cohort studies and meta‐analyses have shown equivalent oncological outcome between transverse colectomy and extended colectomy. It is time to resurrect transverse colectomy and consider it equivalent to its radical counterpart for cancers around the mid‐transverse colon.

## Graphical abstract

Transverse colectomy for transverse colon carcinoma is oncologically equivalent to the more radical extended right hemicolectomy or subtotal colectomy.

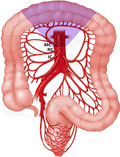

## 1 INTRODUCTION

**FIGURE 1:**
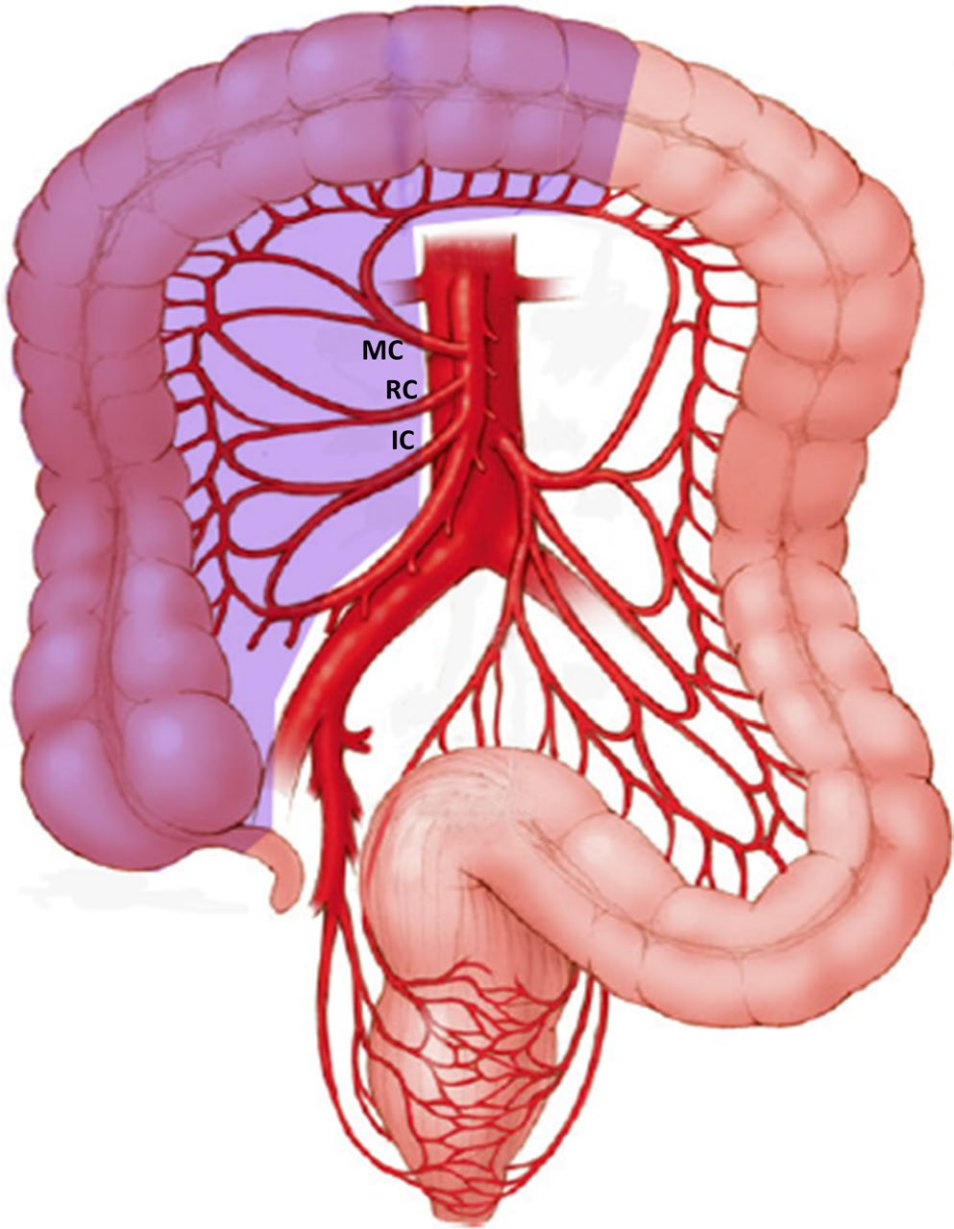
Extended right hemicolectomy (IC: ileocolic artery, RC: right colic artery, MC: middle colic artery; shaded area: extent of colonic resection)

**FIGURE 2:**
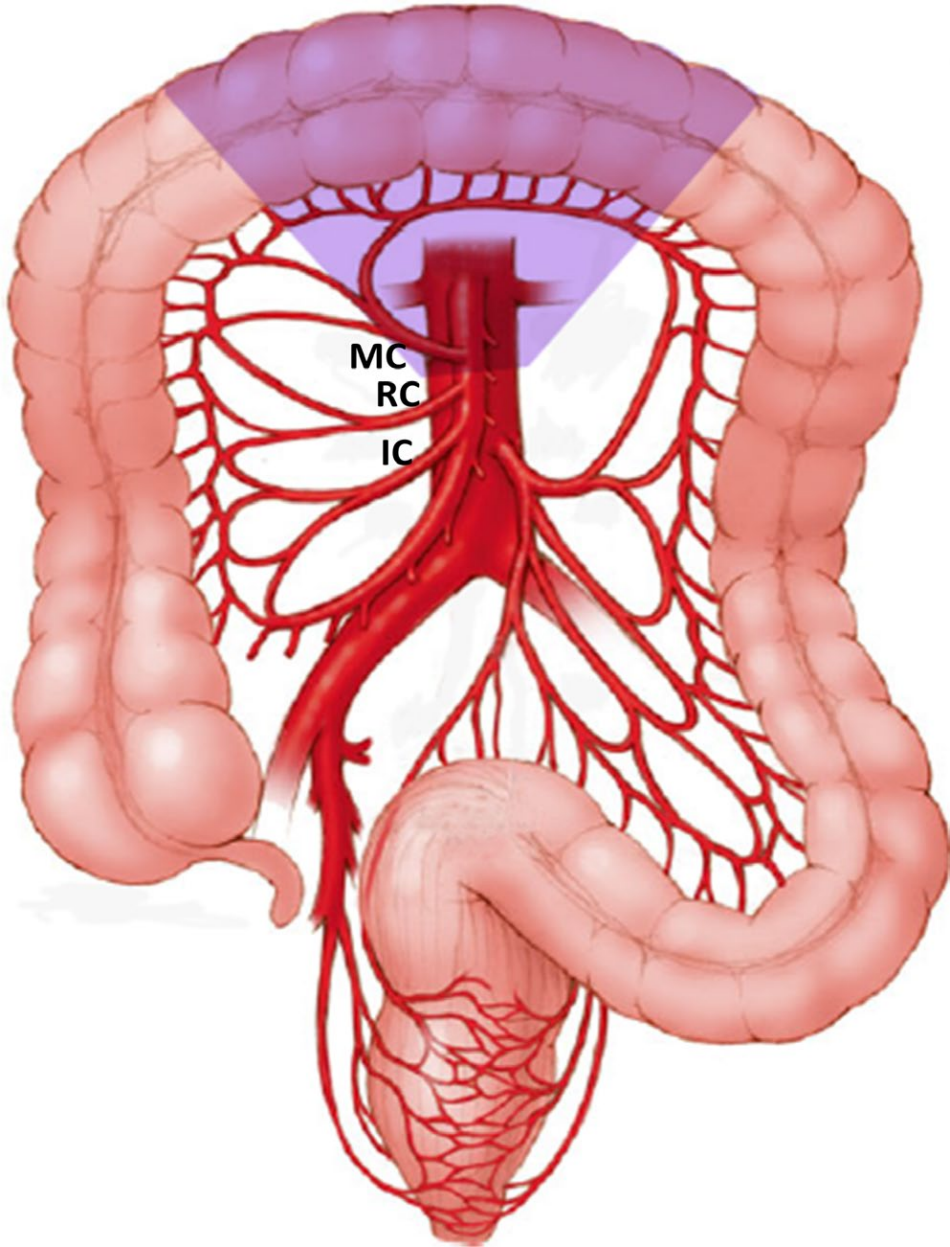
Transverse colectomy (IC: ileocolic artery, RC: right colic artery, MC: middle colic artery; shaded area: extent of colonic resection)

**FIGURE 3:**
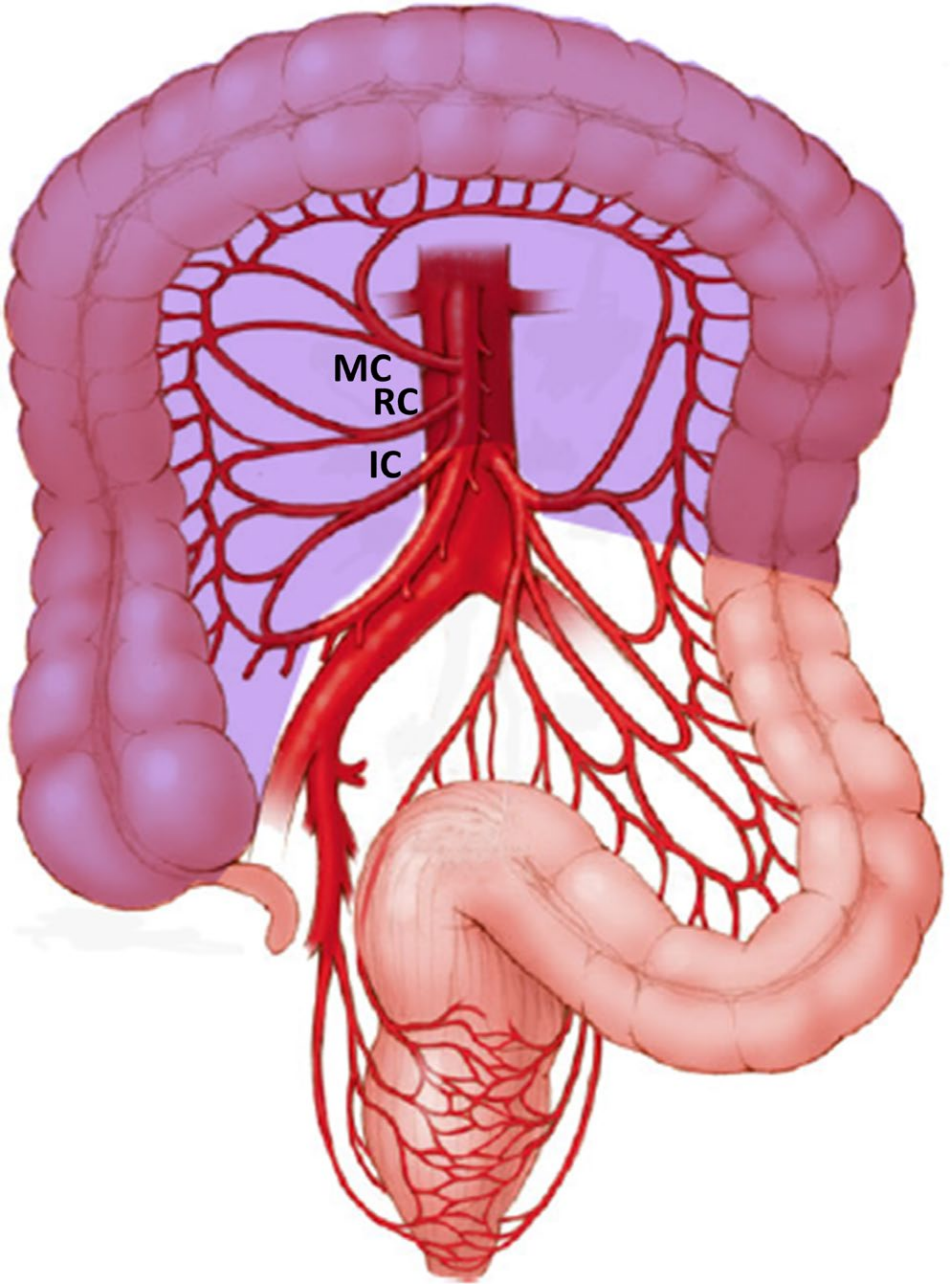
Subtotal colectomy (IC: ileocolic artery, RC: right colic artery, MC: middle colic artery; shaded area: extent of colonic resection)

The transverse colon, the longest segment of the large intestine, is rather unique. With the caecum and right colon, it arises primarily from the midgut and its mucosa is exposed to a similar concentration of biliary salts and bacterial composition as the right colon. But, despite its mucosal surface area being about 2.5 times that of the right and sigmoid colon, the age and mucosal‐surface standardized incidence rate of transverse colon cancer is the least among other sites of colon.^1^ In terms of gross pathology, only 10% of colonic cancers arise in the transverse colon.^2^, ^3^ As its proximal two‐third is derived from the midgut while the distal one‐third is hindgut in origin, these two segments are supplied by middle colic and left colic artery, respectively. Hence lymphatic spread of transverse colon adenocarcinoma may occur in different directions. This coupled with the fact that the transverse colon is completely intraperitoneal, covered with greater omentum, situated close to liver, stomach, pancreas and spleen, and whose operations entail mobilization of the hepatic and splenic flexures, means that surgery for transverse colon cancer poses considerable challenges. While there is no ambiguity that hepatic flexure adenocarcinoma should be treated with extended right hemicolectomy (Figure 1),^4^ splenic flexure cancers have been resected by left hemicolectomy, segmental colectomy, and subtotal colectomy (Figures 2 and 3).^5^, ^6^, ^7^ For all these reasons, transverse colon cancers had been excluded from the prospective randomized trials comparing laparoscopic with open colectomy.^8^, ^9^, ^10^ This review focuses on the literature surrounding the surgical treatment of transverse colon cancers as there is are no unanimity about the extent of colonic resection and nodal clearance.

## 2 LITERATURE REVIEW

In one of the earliest publications in 1939, Mayo et al presented their experience in treating 204 patients with transverse colon cancers between 1907 and 1936.^2^ Among these, 95 (46.6%) patients underwent extraperitoneal resection, 36 (17.6%) patients had resection and anastomosis, while resection of right half of colon, transverse colon and ileocolostomy was performed in 16 (7.8%) patients. The hospital mortality in the second and third group was 11.1% and 43.7%, respectively. With the adoption of the Halstedian model of cancer progression from the primary to the lymph nodes along the draining vessels, colectomy became more radical, but survival of transverse colon cancer patients continued to be poor.^11^ Standard textbooks advocate transverse colectomy with the ligation of middle colic pedicle as the treatment of choice for such cancers.^12^ However, it has also been managed by extended right hemicolectomy and subtotal colectomy (Figures 1, 2, 3).^13^, ^14^, ^15^, ^16^ Part of the confusion in the published literature is because many studies have included hepatic and splenic flexure cancers, and there is paucity of data regarding the treatment of transverse colon cancers away from the flexures.

Rongen et al reviewed their experience with 103 patients with transverse colon cancers who were treated between 1989 and 2003.^16^ Extended colectomy (EC) was performed in 69 (67%) patients while 34 (33%) underwent transverse colectomy (TC). The hepatic flexure was mobilized in 20%, splenic flexure in 18%, while in 1% of patients both the flexures were mobilized. There was no mention about the level of ligation of vascular pedicle, and mean lymph node harvest was less than five in both the groups (Table 1). For TNM stages I‐II, the 5‐year overall survival (OS) was slightly higher for the hemicolectomy group (65% vs 55%) but this was not statistically significant (*P* = .38). Similar survival finding was noted for TNM stage III: 52% vs 42%, *P* = .82. Taking all the stages together, their 5‐year overall survival was 61% for extended colectomy group and 50% for patients who had transverse colectomy (*P* = .34).

**Table 1:** Studies comparing extended colectomy with transverse colectomy

Parameters	Rongen et al^16^	Matsuda et al^15^	Chong et al^13^	Leijssen et al^14^
Extended colectomy (EC)	69	38	127^a^	32^a^
Extended right hemicolectomy	Not mentioned separately	52.80%	70.40%	46.60%
Left hemicolectomy	Not mentioned separately	‐‐	11.90%	16.50%
Transverse colectomy	33%	47.20%	17.70%	36.90%
Transverse colectomy (TC)	34	34	127^•^	32^a^
Laparoscopic approach EC vs TC	No laparoscopy	All laparoscopy	48% vs 25.2%	43.8% vs 34.4%
Number of nodes EC vs TC	3.4 vs 4.5^b^	26 vs 12^c^	23 vs 16^c^	25 vs17^c^
*P* value	.2 (NS)	.000 (S)	<.001 (S)	<.001 (S)
Post‐op complications EC vs TC	‐‐‐‐‐‐	10.5 vs 29.4	26.8 vs 19.7	43.8 vs 40.6
*P* value		.014 (S)	.038 (S)	1.00 (NS)
Anastomotic leakage EC vs TC	2.9% vs 2.9%	0% vs 5.8%	2.4% vs 0.8%	3.1% vs 3.1%
*P* value	1 (NS)	.13 (NS)	.62 (NS)	1 (NS)
*P* value		.593 (NS)	.128 (NS)	.05 (NS)
5 years OS EC vs TC	61% vs 50%	90.3% vs 79.6%	86.6% vs 84.3%	81.3% vs 78.5%
*P* value	.34 (NS)	.638 (NS)	.282(NS)	.418 (NS)

Abbreviations:

DFS, disease free survival; NS, statistically not significant; OS, overall survival; S, statistically significant.

^a^ Number of patients after propensity score matching.

^b^ Mean.

^c^ Median.

Matsuda et al compared their experience with laparoscopic EC (38 patients) vis‐à‐vis laparoscopic TC (34 patients) performed between January 2007 and April 2017 for mid‐transverse colon cancers.^15^ In the EC arm, all underwent extended right hemicolectomy. The mid‐transverse colon was defined as the middle one‐third of the transverse colon. During transverse colectomy only partial colonic resection was performed. The operations were performed with D2 or D3 lymphadenectomy and adhered to the Japanese Society for Cancer of the Colon and Rectum Guidelines.^17^ The cT1/T2 tumours, with no nodal disease, are dealt with D2 transverse colectomy during which the right and left branch of middle colic artery are ligated separately and the middle colic trunk is spared. During this the para‐ and pericolic nodes and intermediate nodes are removed. D3 dissection, which in addition removes the main nodes, is performed for cT3/T4 disease and/or nodal metastases. This entails ligation of ileocolic, right colic, and middle colic artery at their respective origin during extended right hemicolectomy, while for transverse colectomy this would mean ligation of the main trunk of middle colic artery. There was no difference in the incidence of T3/T4 cancers in either group but average nodal harvest was 26 vs 12 (*P* = .000). There was no postoperative mortality. The 5‐year disease free survival (DFS) (92.4% vs 95.7%, *P* = .593) and 5‐year OS (90.3% vs 79.6%, *P* = .638) were similar between the two groups. In the extended right hemicolectomy group, there was no nodal metastasis along the right colic or the ileocolic vessels; the single patient with nodal recurrence developed metastases in the para‐aortic and supraclavicular nodes. Despite the omission of lymphadenectomy along the right colic vessels, the transverse colectomy group did not develop any local recurrence. It is an interesting finding as the right colic and middle colic artery may arise as a common trunk in 4% of the population^18^ and is in contrast to the study by Park et al who found metastases in right colic nodes in 10% of their 58 patients with transverse colon cancer.^19^

In another study involving 1006 patients operated on between 1995 and 2013, Chong et al compared the results of EC with TC.^13^ The tumour location ranged from the hepatic to splenic flexure. In total, 939 (88%) patients underwent EC (extended right hemicolectomy in 750 patients and left hemicolectomy in 189 patients), while TC was performed in 127 (12%) patients. Patients who had extended right hemicolectomy underwent ligation of ileocolic, right colic, and middle colic vessel ligation at their origin, while during left hemicolectomy the left colic pedicle and either the left branch of the middle colic or the origin of the middle colic pedicle were ligated. During transverse colectomy the middle colic pedicle was ligated at its origin. A minimum of 5 cm was adopted for colonic resection margin. After propensity score matching there were 127 patients in each group. The T3/T4 patients constituted 63% in both the arms. The median specimen length was 34 cm for the EC group and 19 cm for the TC group, while the median number of total nodes retrieved was 23 and 16, respectively. At a median follow‐up of 59.6 (0.5‐242) months, the 5‐year DFS (85% vs 89.8%, *P* = .128) and 5‐year OS (86.6% vs. 84.3%, *P* = .282) was not different between the two groups. The finding that the length of the resected bowel and number of nodes did not influence the survival has also been one of the conclusions of the COST study.^20^ Central vascular ligation was performed routinely and the study reported a local recurrence rate of 1.4% and 0.8%, respectively. In this extended study, 13% patients were lost to follow‐up.

The Surveillance, Epidemiology, and End‐Results (SEER) database was interrogated by Guan et al between 2004 and 2013.^21^ This database included 17 registries covering approximately 28% of the American population. The study identified 10 344 patients with transverse colon cancer. EC was performed in 5913 (57.2%) patients while 4431 (42.8%) patients underwent TC. Among the patients who had EC, 80.3% had more than 12 nodes, the minimum number of nodal harvest recommended by most guidelines,^22^ removed while this criteria was met in 62% patients who underwent TC. This is concordant with other studies. Although the nodal harvest was less in TC, the incidence of nodal metastases was 34.4% in both the groups. The 5‐year cancer specific survival was 66.5% in patients who underwent EC while it was 67.5% for those who had TC (*P* = .170). Propensity score matching revealed similar results. However, subgroup analysis revealed that for the larger tumors (>5 cm), there was a survival advantage following extended colectomy (HR: 1.136, 95% CI: 1.055‐1.222, *P* = .001). As this study identified patients from a registry, information regarding postoperative complications, surgical technique (laparoscopy and open), and short‐term results were lacking.

Leijssen et al published their experience with 103 patient with mid‐transverse colon cancer treated between 2004 and 2014.^14^ There were 65 (63.1%) patients in the EC group (extended right hemicolectomy in 48 patients, left hemicolectomy in 17 patients) while TC was performed in 38 (36.9%) patients. To minimize the impact of confounding in this retrospective study, propensity score matching was performed, which led to 32 patients being in each treatment arm. Around 40% patients had all‐cause complications in both the groups. The postoperative mortality was 3.1% in patients who had TC but none of the patients who underwent EC died. The tumour and nodal stage was also equally distributed, but the number of nodes harvested was more in patients who had EC (median: 25 vs 17, *P* < .001). In both the groups, 84.4% had more than 12 nodes harvested. Patients who underwent TC had tumours with worse histological parameters like poor differentiation (0% vs 15.6%), perineural invasion (0% vs 9.4%), and stable microsatellite instability (6.3% vs 21.9%). However, the 5‐year OS (81.3% vs 78.5%, *P* = .418) and 5‐year DFS (100% vs 84.9%, *P* = .05) was similar in both the treatment groups at a median follow‐up of 48.6 (19.5‐73.5) months.

The postoperative bowel function has been studied in some studies. Matsuda et al noted that the time to first flatus (median: 2 days) and time to resumption of liquid diet (median: 4 days) was similar in both the treatment arms.^15^ Similarly, Leijssen et al found that postoperative ileus developed in 12.5% patients in both the groups.^14^

These retrospective studies have extended over a long period and have witnessed the introduction of minimally invasive techniques. Among the above studies, Matsuda et al had performed all the operations laparoscopically.^15^ In the series by Chong et al^13^ and Leijssen et al^14^ around one‐third of the procedures were performed by laparoscopic approach. These two studies also noted an increasing trend of laparoscopic surgery and decreasing numbers of transverse colectomy in the latter half of the study period. Since the first report of laparoscopic colectomy in 1991,^23^ laparoscopic colonic resections gained popularity in the early 2000s. During the initial part of the learning curve, laparoscopic dissection of the middle colic pedicle and mobilization of both the flexures required during transverse colectomy were perceived as difficult steps. Also there was a notion that the increased length of colonic resection and consequently increased nodal harvest, which is so with extended colectomy, will translate into better survival. These factors contributed to laparoscopic extended colectomy becoming a much more popular choice in treating transverse colon cancers than laparoscopic transverse colectomy. However, as is clear from the above studies (Table 1), there is survival equivalence between extended colectomy and transverse colectomy.

The wide spread acceptance of laparoscopy has led to studies comparing the short‐ and long‐term results of laparoscopic vs open operations for transverse colon cancers. Most of the studies have originated from South and Far East Asia, are retrospective in nature and have small number of patients. As a result, and in order to garner evidence, these studies have been subjected to meta‐analysis. To date, five such meta‐analyses have been published (Table 2).^24^, ^25^, ^26^, ^27^, ^28^ These meta‐analyses have spanned an overlapping time period but the studies included have differed. This is because of varying inclusion and exclusion criteria used by the respective authors. Due to small numbers of patients in each study and differences among the population, a number of parameters studied in these meta‐analyses showed marked heterogeneity. Despite these limitations, the intra‐operative blood loss, time to passage of first flatus, time to resumption of oral diet, and length of hospital stay was less in patients who underwent laparoscopic colectomy. This was offset by increased operative duration in this group. However, the postoperative morbidity and mortality were similar. There was no difference in the number of nodes harvested in each of the procedures. The 5‐year disease‐free and overall survival were also equivalent.^25^, ^27^, ^28^ However, the quality of evidence is dilute as the studies were retrospective and had small numbers of patients. Many of the studies included tumours over both the flexures, while some had pure transverse colon cancers. Thus, although randomized controlled trials would be the natural choice to generate higher levels of evidence, due to the improved postoperative recovery following laparoscopic operations, randomization will not be ethical and controlled prospective trials need to be performed to provide the answer.

**Table 2:** Summary of meta‐analysis comparing the short‐ and long‐term outcomes of laparoscopic versus open colectomy for transverse colon cancers

Author	Study period	Number of studies	Number of laparoscopic colectomy	Number of open colectomy	Short‐term outcomes	Long‐term outcomes
Chand et al (2014)^24^	Till 2013	5	245	199	Similar intra‐operative blood loss, post‐op morbidity and mortality and number of nodes harvested. Increased operative duration, but early passage of first flatus, time to resumption of oral diet and decreased length of hospital stay in laparoscopic colectomy.	Equivalent 5‐years disease free and overall survival (^25^, ^27^, ^28^)
Athanasio et al (2017)^25^	1990‐July 2016	11	763	652		
Wu et al (2017)^26^	2010‐Sep 2016	13	894	834		
Gavriilidis et al (2018)^27^	Last 20 years	8	546	401		
Baloyiannis et al (2020)^28^	2009‐2019	21	1254	1244		

As mentioned above, many publications have included splenic flexure tumours when analyzing outcome measure of transverse colon cancers. The splenic flexure cancers have been dealt with a variety of operations, viz. extended right colectomy, left hemicolectomy, segmental colectomy, and subtotal colectomy.^5^, ^6^, ^7^ Each of these operations may have their own oncological indications but in reality it is often dictated by the surgeon's preference and experience. A recent retrospective multicentre European study analyzed the surgical and oncological outcomes of 399 splenic flexure cancer patients who underwent extended right colectomy, segmental colectomy, and left hemicolectomy between 2000 and 2018.^29^ An increase in operative time, time to flatus, time to regular diet, and hospital stay was observed in patients who had extended colonic resection as compared to segmental colectomy but the 5‐year overall survival (66.2% vs 76.3% vs 74.3%, *P* = .26) and 5‐year disease‐free survival (73.9% vs 70.3% vs 76.3%, *P* = .56) were similar across the three groups.

In a study published in 1991, the postoperative mortality following transverse colectomy was 10%.^30^ Since then surgical techniques and technology have evolved and a postoperative mortality of 0.4% was noted among the 763 patients who had laparoscopic colectomy in the meta‐analysis by Athanasio et al.^25^ Laparoscopic mobilization of both the flexures is a difficult manoeuvre. With increasing experience, surgeons have got accustomed to the lack of optimum in‐optical axis manipulation and changing azimuth angle faced during laparoscopic colectomy.^31^ Another challenge encountered during laparoscopic transverse colectomy is performing central ligation of the middle colic pedicle. Several techniques to perform this crucial step have been described.

## 3 SURGICAL TECHNIQUES

**FIGURE 4:**
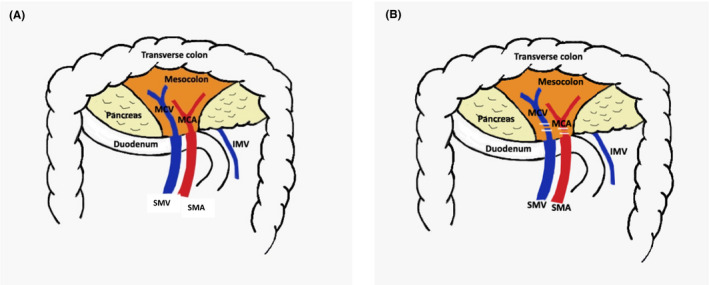
(A) Caudal approach to middle colic pedicle. (B) Caudal approach to ligation of middle colic pedicle (SMV: superior mesenteric vein, SMA: superior mesenteric artery, MCV: middle colic vein, MCA: middle colic artery, IMV: inferior mesenteric vein)

In the medial‐to‐lateral technique by Fujita et al, the transverse colon is lifted up and the ventral aspect of the caudal portion of the superior mesenteric vein (SMV) is exposed^32^ (Figure 4A,B). Further cranial dissection over this vein brings the surgeon to the origin of the middle colic vein (MCV) which is then divided. The superior mesenteric artery (SMA) is exposed on its left side and the origin of the middle colic artery (MCA) dissected and divided.

**FIGURE 5:**
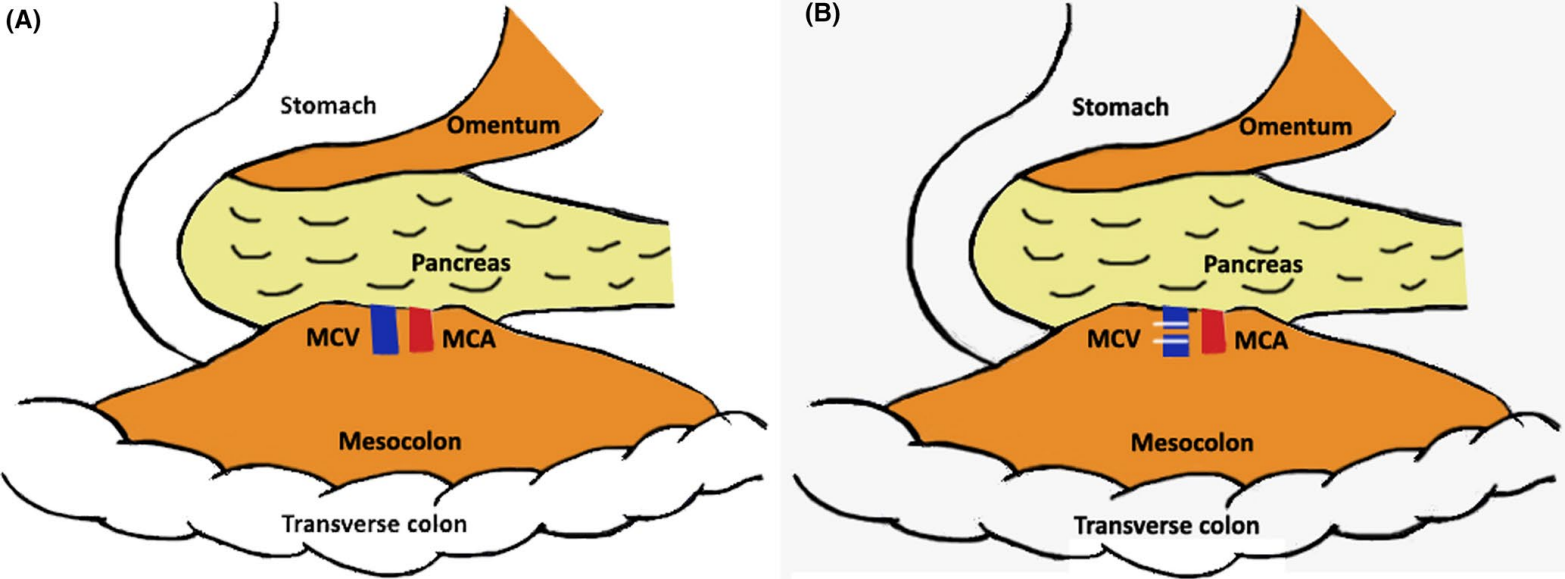
(A) Lesser sac approach to middle colic pedicle. (B) Lesser sac approach to ligation of middle colic vein (MCV: middle colic vein, MCA: middle colic artery)

The MCV can also be approached by incising the omental bursa and entering the lesser sac^33^, ^34^ (Figure 5A,B). The superior right colic vein (SRCV) running towards the gastrocolic trunk is identified and divided. Caudal retraction over the transverse mesocolon (TMC) brings the MCV to view. The TMC is then dissected from the lower border of pancreas and MCV is divided. After this, the transverse colon is retracted cranially and the SMA is identified in the inframesocolic area. The origin of MCA is dissected and divided. In 12% of patients, the MCV drains in to the gastrocolic trunk which, in this technique, is dealt early on.^35^

The “pincer technique” developed by Egi et al is a slight variation of the above technique.^36^ The surgeon starts by standing on the right side of the patient, separates the greater omentum from TMC and enters the lesser sac. The root of TMC is freed from the inferior border of the pancreas thereby identifying the MCV. Dissection then shifts to the inframesocolic area, an incision is made over the TMC lateral to the duodenojejunal flexure and TMC is mobilized from the inferior border of pancreas. Now the surgeon stands on the left of the patient and dissects the right side of the root of TMC to separate it from duodenum and pancreatic head. After this, the surgeon stands between the legs and dissects and divides the MCA and MCV. Finally, the surgeon goes back to the right side of the patient to complete mobilizing colonic attachments and dividing aberrant vessels like accessory middle colic artery (AMCA). The AMCA, present in 36% of population,^37^ arises from the SMA underneath the pancreas and supplies the splenic segment of transverse colon. It is best dealt with as it exits the inferior pancreatic border. The early mobilization of the root of TMC from the pancreatic border allows for developing plenty of space around the vascular pedicles, which are dealt at the end. This strategy of creating wide space surrounding the pedicles has also been utilized by Koinuma et al who studied virtual surgical anatomy using preoperative 3D‐CT and familiarized the surgeons about the vascular variations.^38^

Recently these operations have been performed using the robot, and small cohort studies have been published.^39^, ^40^, ^41^ They underscore the feasibility of this new procedure and suggest that the robot may minimize the necessity of mobilizing both colonic flexures and facilitate intracorporeal hand‐sewn anastomosis.

## 4 SUMMARY

Transverse colon cancers have been managed with extended right colectomy, left hemicolectomy, subtotal colectomy, and even with limited transverse colectomy. This article reviewed the literature regarding the surgical treatment of transverse colon cancer away from the flexures. There is limited high‐quality literature on this primarily because this is an uncommon location for a cancer. Nonetheless, central ligation of the middle colic pedicle and meticulous dissection of the transverse mesocolon followed by a segmental transverse colectomy appears to be an oncologically adequate operation. With the plethora of high‐quality laparoscopic and haemostatic devices available, these operations could be routinely performed by minimally invasive techniques, which ask for proper training, credentials, and experience. This literature review also suggests that future publications should clearly mention the exact location of the tumor. The surgical community should strive towards standardizing the definitions of extended right colectomy, transverse colectomy, and left hemicolectomy and lay down guidelines regarding adopting a particular technique.

## CONFLICTS OF INTEREST

The authors declare no conflicts of interests for this article.
